# Evaluating the impact of a health hackathon on collaborative team science: a Social Network Analysis (SNA)

**DOI:** 10.1017/cts.2020.46

**Published:** 2020-05-18

**Authors:** Layla Fattah, Janice Gabrilove, Fay Bradley

**Affiliations:** 1 ConduITS, Icahn School of Medicine at Mount Sinai, New York, NY, USA; 2 The Tisch Cancer Institute, Icahn School of Medicine at Mount Sinai, New York, NY, USA; 3 University of Manchester, Manchester, UK

**Keywords:** Social Network Analysis, SNA, team science, transdisciplinary, innovation

## Abstract

The Mount Sinai Health Hackathon is designed to provide a novel forum to foster experiential team science training. Utilizing a Social Network Analysis survey, we studied the impact of the Mount Sinai Health Hackathon on the nature of collaborative relationships of hackathon participants. After the event, the number of links between participants from different disciplines increased and network density overall increased, suggesting a more interconnected network with greater interdisciplinary communication. This social network approach may be a useful addition to the evaluation strategies for team science education initiatives.

## Introduction

The Mount Sinai Health Hackathon is an experiential educational event designed to foster collaborative working between participants from a range of disciplines. Launched in 2016, the Mount Sinai Health Hackathon offers an innovative and novel model for team science education and entrepreneurship. Borrowing this format from the technology world, the Mount Sinai Health Hackathon brings together individuals to form interdisciplinary teams with the aim of creating an innovative technology to solve a current problem in medical science or healthcare. Participants form self-selected teams of between 3 and 10 participants and work together over a 48-h period to produce a healthcare-focused technology solution. Teams pitch their ideas to a panel of judges, and three finalists are selected to win a cash prize to support the ongoing development of their prototype technology.

The Mount Sinai Health Hackathon integrates diverse capabilities and domain expertise of individuals from a wide-range of disciplines including basic science, engineering, clinical, bioinformatics, product design and business. As such, one of the main educational aims for the Mount Sinai Health Hackathon is to bring participants together in transdisciplinary teams around a shared problem, fostering experiential learning through communication, collaboration and problem-solving. Bringing participants from diverse disciplines together over a shared problem offers an experiential forum through which to foster transdisciplinary teamwork. Furthermore, integrating participants from different backgrounds provides access to the tacit knowledge of each individual, which is thought to create an environment that promotes innovation [1].

To understand the patterns of collaborative relationships, and specifically interdisciplinary relationships at the Mount Sinai Health Hackathon, we performed a Social Network Analysis (SNA) of interactions between participants at the 2018 Mount Sinai Health Hackathon.

SNA is a quantitative, descriptive research technique that focuses on relationships among people and within groups [2]. A network is a set of nodes and links that connect the nodes. Nodes typically represent people, in this case health hackathon participants. Links indicate a tie or relationship, such as communication or a shared activity. Network analysis uses mathematical equations to measure the network and quantify the relationships among the nodes (for example, the number of links shared between nodes). Relationships between nodes can also be visualized as a network sociogram [2]. A sociogram is a graph that depicts nodes, which are represented by labeled or colored icons, and the links or ties that connect these nodes, which are represented by lines. SNA can be applied to assess collaboration as a function of the pattern of social ties within and among groups. Thus the group, rather than individual members, is the unit of study. This method has been applied across diverse fields of study. In clinical and translational fields of inquiry, SNA has been used to examine primary care practices, community–academic partnerships, institutional culture, and collaborative research practices [3–7].

To date, few studies have measured connections between participants or changes to the diversity of professional networks in a team science educational event, such as a health hackathon.

## Materials and Methods

### Design

Characteristics of the social network between Mount Sinai Health Hackathon participants were measured using a single SNA survey administered directly following the Mount Sinai Health Hackathon in October 2018. Participants were asked to report their collaborations at the event and indicate if they had interacted with this individual prior to participation in the event. The Mount Sinai Institutional Review Board determined this study exempt (HS#: 18-01014). In the introductory section of the questionnaire, potential participants were provided with a written explanation of the project, the human subjects’ protections in place against undue coercion, and breaches in confidentiality and anonymity. They were informed that their participation in the survey was completely voluntary. To protect respondent confidentiality, data were linked to nonsense codes. To avoid re-identification by the research team, the unidentified files and original files were stored on a password-protected computer.

### Survey

The survey contained demographic questions to document age, ethnicity, professional background and organizational affiliation of respondents, as well as the social network instrument. The instrument was originally developed using input from SNA experts at the University of Manchester and the University of Kentucky. The survey was piloted with a small cohort of post-graduate students.

To complete the SNA portion of the survey, the participants reviewed a roster of all health hackathon participants (*n* = 76) and were asked to respond to the following questions for each:

- Did you know this person before participating at this event? (Yes or No)
- Did you collaborate with this person at the hackathon? (Yes or No)
- How important was this person to your hackathon experience? (1 = not at all important; 10 = very important)

The survey took approximately 15 min to complete; responses were imported and analyzed with UCINET network analysis software programs [8].

### Analysis

Three measures of network quality were examined pre- and post-hackathon:

- Network density: a measure of network cohesiveness, which represents the number of links that exist between individuals in relation to all links that could possibly exist in the network
- E-I index: a measure of the relative density of internal connections within a social group compared to the number of connections to an external group
- Degree centrality: a measure of how connected and potentially influential a participant is within the network. This is the number of ties a node has when compared to other nodes in the network.

Finally, network sociograms were generated for both time points using Netdraw software. These diagrams, in which nodes are colored to represent identifiers, such as participants from different professional backgrounds, allowed visual inspection of the patterns described by the network measurements.

## Results

Table 1 shows characteristics of SNA responders. Sixty-four percent (49/76) completed the SNA portion of the survey.

**Table 1.** Demographics of Social Network Analysis survey respondents

Age	18–25 years: 63% 26–33 years: 28% >33 years: 9%
Gender	Female: 43% Male: 57%
Professional background	Bioinformatics: 24% Clinical: 25% Basic Science: 6% Product Design: 3% Engineering: 16% Software: 22% Business: 3%

**Fig. 1.**
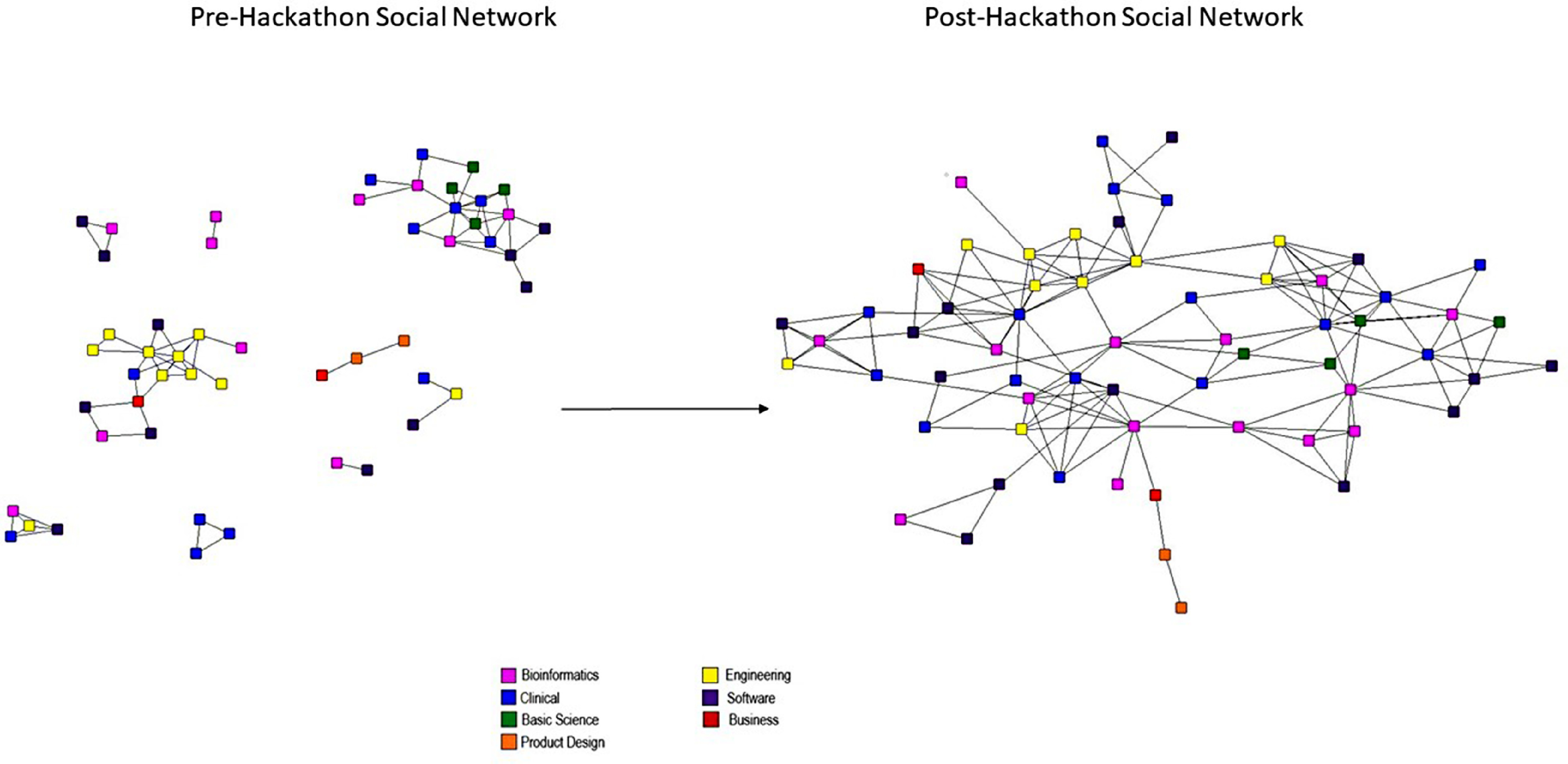
Sociogram of the Mount Sinai Health Hackathon participants: pre- and post-hackathon.

The network diagrams between participants pre- and post-hackathon are displayed in Fig. 1 and measurements are given in Table 2. At baseline, the density score was low (0.12), indicating a relatively low number of connections between participants in this network pre-hackathon. The E-I index based on professional backgrounds was 0.26, indicating a slight preference for connection to participants from other disciplines, when compared with connections to participants from the same discipline. Visual examination of the sociogram in Fig. 1, pre-hackathon, shows a number of isolated groups, consisting mainly of either engineers or clinicians and basic scientists. Post-hackathon, the density score for the overall network had risen to 0.30. At the same time the E-I index had risen to 0.43, indicating an increase in interdisciplinary ties between individuals from different professional backgrounds.

**Table 2.** Network measurement results pre- and post-hackathon

	Pre-hackathon	Post-hackathon
Network density	0.12	0.30
E-I index	0.26	0.43

**Fig. 2.**
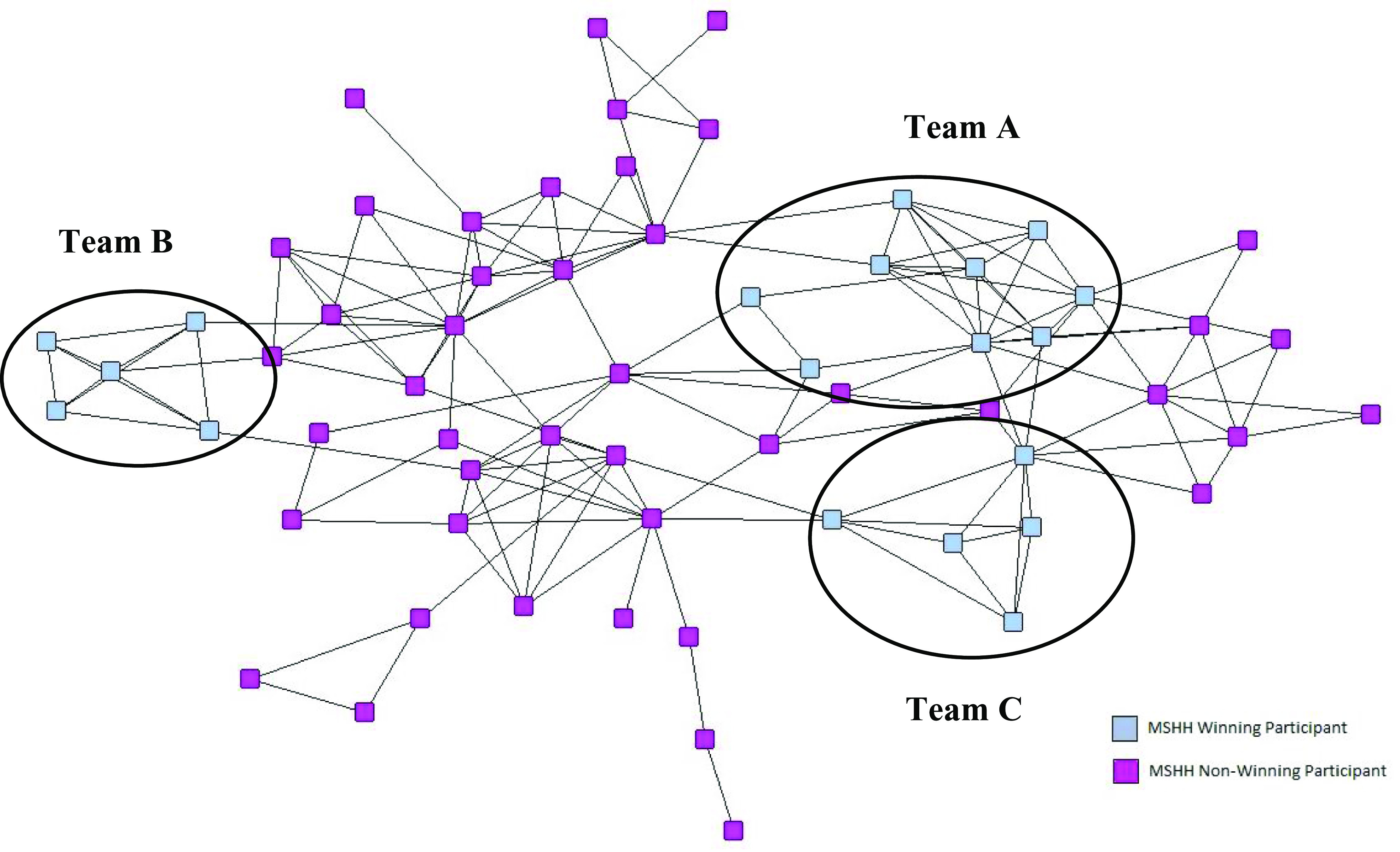
Sociogram of the Mount Sinai Health Hackathon participants: winning teams.

Degree centrality was calculated for each of the individual participants and the mean degree centrality calculated for the overall network, and for each of the three winning teams. The network diagram in Fig. 2 depicts the winning participant teams and Table 3 shows the mean degree centrality for the winning teams in relation to the overall network.

**Table 3.** Mean degree centrality for the overall network and winning teams at the Mount Sinai Health Hackathon

	Mean degree centrality (range)	Number of team members (number of professions represented)
Overall network	4.1 (0–12)	Mean 4.9 (2.9)
Winning Team A	6.6 (3–11)	9 (5)
Winning Team B	4.8 (4–6)	5 (4)
Winning Team C	5.6 (4–10)	5 (2)

The degree centrality scores of participants on the winning teams ranged from a relatively low degree centrality of 3 (indicating the participant is only connected to their direct team members) to a relatively high degree centrality of 11 (indicating the individual is connected to members of the other teams). All three winning teams had a mean degree centrality higher than the mean of the overall network.

## Discussion

One of the main aims of the Mount Sinai Health Hackathon is to enhance transdiscplinary collaborations between individuals from diverse professional backgrounds. The findings suggest that the network became more interconnected and patterns of interaction became more interdisciplinary as measured by a SNA approach.

At baseline, the network was less dense. Participants had fewer pre-existing professional relationships, and were less likely to be connected to participants from a different professional background. Post-hackathon we found an increase in network density, indicating an overall increase in ties between participants, and an increase in the E-I index, indicating that relationships were increasingly formed between participants from different disciplines. The participants were free to select their own teams; so the interdisciplinary nature of these teams suggests that participants saw value in collaborating with individuals from different professional backgrounds. It is equally interesting to note that although the participants worked in self-selected teams of 3–10 participants, the network is joined, with at least one participant from each team reporting a relationship with an individual external to their team. There are several theories on how network data is linked to innovation and one of these theories posits that weak ties with individuals not in one’s direct network may lead to increased innovation [9,10]. In this context, new information is obtained through relationships to actors who are not members of the closely connected part of the network. There are individuals within the network that act as bridges, allowing information to be transferred between teams [11]. This connected network that allows for sharing of ideas between teams is an interesting feature of this network.

In terms of team success, whilst there was no formula for winning a prize at the health hackathon, there were common themes among the winners. All the winning teams had a high mean degree centrality, indicating participants in these teams were well-connected within the network. It should be noted that team A had higher than average number of team members, which means the degree centrality of these team members is likely to be higher than the mean. However, these were self-selecting teams and therefore made these connections themselves. Teams B and C were of average size, but still had higher than average degree centrality. Winning teams A and B were professionally diverse with four or more professions represented. Team C had only two professions.

Based on this data it is not possible to make any correlation between team network data and team success. However, it is interesting that three of the six individuals with the highest degree centrality were on winning teams, and all the teams has at least one team member who was more well connected than the mean. Participants with high degree centrality are potentially influential, as they have greater opportunity to be sources or recipients of information from others. These individuals were potentially the ones who were able to foster information and idea sharing between their team and other teams [12]. The transfer of information through these individuals allows knowledge to be shared and recombined, a process which is essential for the creation of new innovations [13].

Our study has several limitations. Firstly, although 64% of the participants completed the SNA survey, a portion of the network is not represented in the data, and therefore any conclusion must be tempered with this information. However, in a network such as this where reciprocation is not required, a tie is assumed if node 1 nominates node 2 even without a response from node 2. Here, a 60–70% response rate has been found to provide relatively robust results [14]. Secondly, we used an approach common to other SNAs, but connection data are self-reported and cannot be verified. Thirdly, network density, E-I index and degree centrality are only a subset of potential SNA centrality measures. We selected degree centrality as this identifies individuals who are likely to act as information transmitters across a wider network, and this is particularly relevant to innovation [15]. Our analysis could have been different had we used other measures such as: betweenness centrality (identifies gatekeepers or brokers within a network); Eigenvector centrality (identifies individuals who are highly connected to other well-connected individuals); or closeness centrality (measures the distance between individuals in the network). Additionally, this data is captured at a single time point and collecting longitudinal data would be valuable in determining the sustainability of these connections. Participants were contacted by email 6 months post-hackathon in an attempt to gather follow-up data. Unfortunately, a low response rate (21%) did not allow for meaningful analysis. The team is developing processes to maintain contact with participants through an online communication platform, which, as well as maintaining participant connections, may also serve to improve future follow-up response rates. Finally, the data were collected in 2018 from a single health hackathon event and may not be representative of other health hackathon events. Even so, this approach could serve to inform future studies.

## Conclusion

To our knowledge, ours is one of the first studies to measure connection between participants at a health hackathon event. We used SNA to measure this connection, a method adopted by other fields such as business. Given the high levels of investment in health hackathon initiatives, our study demonstrates a new approach to understand the value and effects of interactions at such events. Future studies could improve upon these techniques via novel methods, such as smart badges that measure human interactions. Future investigation could also explore the qualitative nature of participant relationships, thereby offering insights into the roles these individuals play in their teams. Additionally, there is potential to use a longitudinal SNA approach to determine if relationships have been maintained between participants and further capitalized on. The results of this study, though preliminary, may provide a basis for further investigation to understand how innovation development evolves through the formation of an interdisciplinary network. Information like this could potentially be used to improve the health hackathon experience for organizations and participants alike.
